# Sustainability and social transformation: the role of ecovillages in confluence with the *pluriverse* of community-led alternatives

**DOI:** 10.1007/s44168-022-00022-5

**Published:** 2022-09-09

**Authors:** Renata Amorim Almeida Fonseca, Marta de Azevedo Irving, Yasmin Xavier Guimarães Nasri, Graciella Faico Ferreira

**Affiliations:** Postgraduate Program on Community Psychosociology and Social Ecology (PPG EICOS), Institute of Psychology (IP), Federal University of Rio de Janeiro (UFRJ), Av. Pasteur, 250, Pavilhão Milton Campos, Praia Vermelha, Rio de Janeiro, RJ CEP 22290-240 Brazil

**Keywords:** Sustainability, Ecovillages, Pluriverse, Buen Vivir, 2030 Agenda, Climate emergency, Sustainability

## Abstract

Amidst the uncertainties of a climate emergency scenario, sustainable and counter-hegemonic alternatives of social and productive organization are being developed by several grassroots local communities. Thus, this essay aims to critically discuss the role of ecovillages within the *pluriverse* of community-led alternatives, understood as a plural set of socially and environmentally transformative possibilities. Adopting a decolonial perspective, this study was carried out through an exploratory qualitative approach, based on a bibliographic and documentary survey. According to the adopted premises, it is possible to recognize that communal territorial innovations, as illustrated by ecovillages’ practices, may expand the horizon of solutions to face contemporary multiple crises. In line with the Paris Agreement and the 2030 Agenda, confluences between diverse community-led alternatives can contribute to deconstruct rooted development premises and to potentialize climate actions toward a just and sustainable transition, in tune with the triune dimensions of cultural identity, social equity, and ecological sustainability of the *Buen Vivir* communal ethics.

**Graphical abstract:**

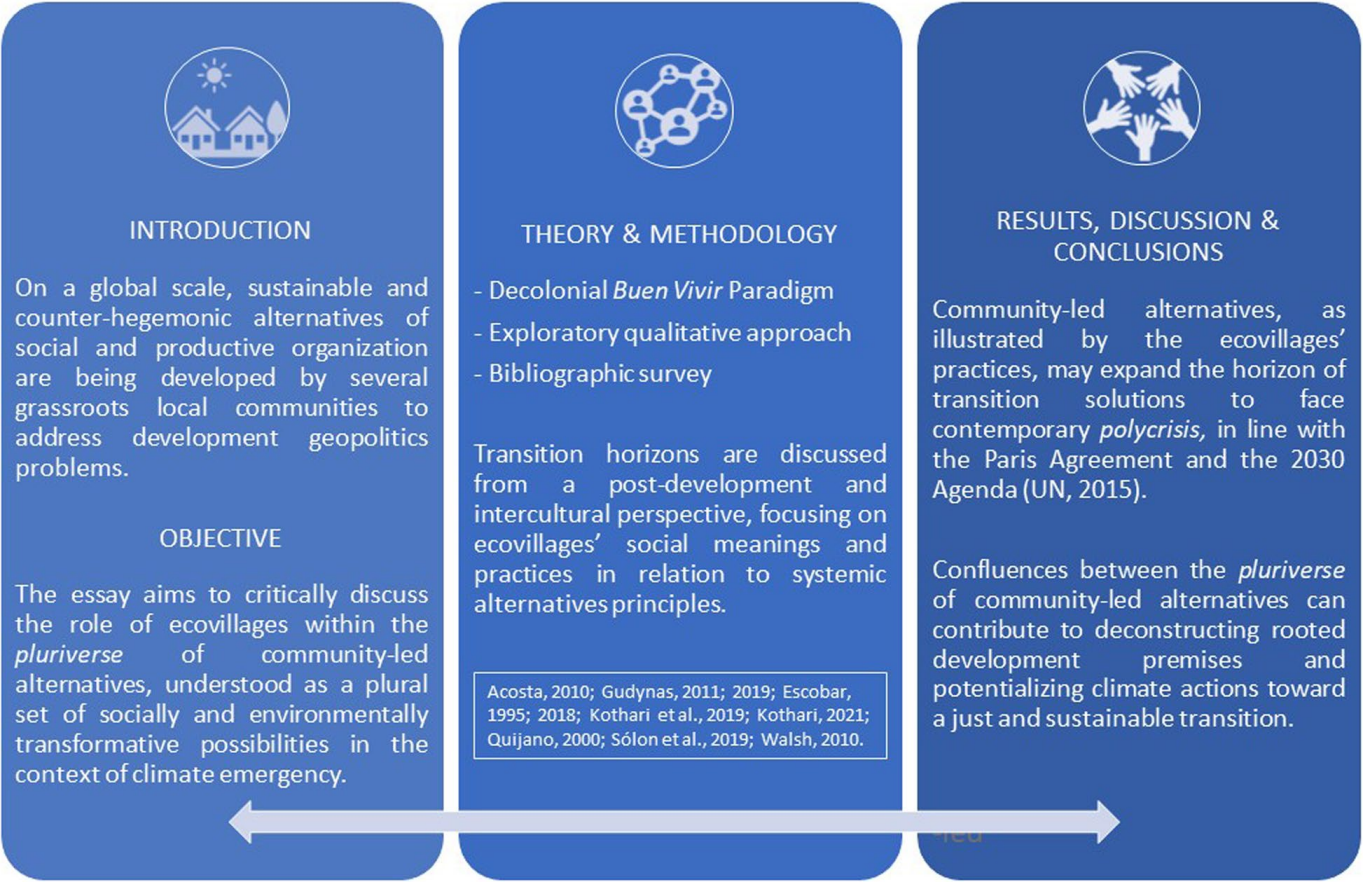

## Introduction

On a global scale, diverse peoples and communities have been facing numerous pressures arising from the development geopolitics, as illustrated by the Covid-19 pandemic reality. As a strategy of resistance and (re)existence, many counter-hegemonic production and social organizations are being built within the *pluriverse*, which is understood as a wide range of social and environmental transformative possibilities, involving a pluralism of concepts, cosmovisions, and practices in tune with the principles of social and ecological justice. *Pluriversal* community-led alternatives are engaged in the construction of “a world where other worlds fit,” according to Zapatista’s principles, in contrast to the ethnocentrism and universalism propagated by Western and modern worldviews (Illich 1973; Escobar 1995, 2018; De la Cadena and Blaser 2018; Kothari et al. 2019).

Connected to their own lifestyles, territorialities, cultures, and social struggles, the confrontation of countless daily challenges, especially by the marginalized minoritarian^1^ social groups (Porto Gonçalves 2002; Haesbaert 2007; Kothari 2021), is generating learning opportunities about resilience and hope that contribute to rethinking the complex scenario of the ongoing transformations, also regarding climate change. Addressing contemporary complexities, inspiring systemic alternatives are being created to face economic, ecological, social, cultural, and ethical-political structural problems (Dilger et al. 2016; Solón et al. 2019; Norberg-Hodge 2019; Kothari 2021). Besides, by being developed within the framework of capitalism itself, these grassroots innovations can thus be understood as “interstitial” and “prefigurative” of a postcapitalist society (Monticelli 2018).

Joining the emergent set of civil society regenerative and localized solutions (Wahl 2019; Norberg-Hodge 2019), the ecovillage movement proposes a feasible whole system design toward sustainable ways of living (Global Ecovillage Network – GEN 2020). Meanwhile embodying potential pathways to promote greater connection with oneself, with others, and with nature itself, ecovillages are being recognized for prefiguring small-scale transition possibilities toward a resilient, equitable, and ecological society (Trainer 2000). Therefore, grassroots initiatives are increasingly receiving attention from the international scientific community, although they are still being neglected by public policies to address such objectives (Roysen et al. 2021).

Ecovillages are configured through autonomous, local, and direct participatory processes, aiming to integrate several dimensions of sustainability (including the ecological, economic, social, cultural) (Gaia Education 2005). But to achieve environmental bioregional regeneration purposes depends also on attempting to the diverse sociocultural and historical backgrounds involved in local dynamics (Dawson 2006; Liftin 2014; Mattos 2018; Roysen and Mertens 2019; Wahl 2019).

Emerging from the “Global North,” as intentional communities guided by sustainability principles, nowadays, ecovillages have acquired new contours with the movement’s transposition to the “Global South,” influenced by traditional communities’^2^ values and ways of living, intrinsically connected to nature’s vital cycles. So, as they are currently spread over all continents and interlinked through the Global Ecovillage Network (GEN), it is important to scrutinize the development geopolitics and the “north-south” relations (Santos 2007) permeating ecovillages’ approaches (Fotopoulos 2000; Silva 2014).

In this context, reflecting on the interconnections between the socio-economic inequalities^3^ and the ecological and climatic collapses, as results of the colonial exploitative socio-historic process, this essay aims to critically discuss the role of ecovillages within the *pluriverse* of community-led alternatives, facing the climate emergency on the horizon of the 2030 Agenda. Based on a Latin American decolonial perspective (Quijano 2000; Castro-Gómez and Grosfoguel 2007), recognizing the coloniality of power that is still being deeply perpetuated by western cultural values, this essay seeks to break through the dominant discourses and to open possibilities for other references, ontologies, and epistemologies beyond the Eurocentric ones. For that, it was inspired by the *Buen Vivir* paradigm (Acosta 2010; Walsh 2010; Gudynas 2011), based on the Kichwa term *sumak kawsay* and the Aymara *suma qamaña*, which is one of the alternatives to modern development brought forward by *pluriverse* literature (Escobar 2018; Kothari et al. 2019; Lang 2022).

From this perspective, the essay is structured in three sections, in addition to this introduction. The first one proposes a brief discussion about the ecovillages as a global movement and the development notion itself. In the second one, transition horizons are discussed from a post-development and intercultural perspective, focusing on ecovillages’ social meanings and practices in relation to systemic alternatives principles of the *pluriverse* of community-led initiatives. A synthesis of this reflection, constitutes the last third section.

## Ecovillages as a global movement and the development paradigm at stake

As briefly introduced, amidst contemporary social and environmental complex challenges, civil society itself is developing innovative solutions aiming to achieve sustainable livelihoods. Among this *pluriverse* of community-led alternatives, there are human settlements known as ecovillages and described as “niches of grassroots innovation” (Roysen and Mertens 2019; Boyer 2015), as well as “demonstrative centers in sustainable practices” (Salazar 2013), or even as “alternative development paradigm” (Veteto and Lockyer 2008). The emergence of these communities in the “Global North” is inspired by the counterculture movements of the 1960s/1970s as alternative ways of living in moments of crisis, gathering those people who did not identify with the dominant ways of thinking-being (Dawson 2013; Silva 2014; Santos-Júnior 2016; Roysen et al. 2021).

But since its origins, in the 1990s (Gilman and Gilman, 1991), the ecovillage concept has undergone important redefinitions, especially in recent years, when traditional communities and their perspectives were encompassed by the proposal. Nowadays, ecovillage is institutionally defined by the Global Ecovillage Network as “intentional, traditional, or urban community that is consciously designed through locally owned participatory processes in all four dimensions of sustainability (social, culture, ecology, and economy) to regenerate social and natural environments” (Global Ecovillage Network – GEN 2022).

So, the ecovillages’ movement encompasses heterogeneous projects, developed by a plurality of social groups expressing different subjectivities concerning community and nature. According to Dawson (2006), on the margins of capitalism, ecovillages, in general, aim to rescue ancestral values and local practices threatened by modernity, while at the core of the capitalist society, they stand out due to their pursuit of lifestyle changes, human values rescue, reconnection with nature, and, above all, with each other. Joubert and Alfred (2007) reaffirm this perspective, emphasizing the communal ties reconstruction as a central aspect for mitigating environmental impacts on ecovillages in the “Global North.” In turn, ecovillages in the “Global South,” according to these authors, seek improvements in life conditions based on solidary relationships and on the sense of belonging to nature, experienced not as a resource but, actually, as a living organism that represents the mother of the all-embracing interconnected community from whom everyone depends.

Whereas neoliberal capitalism advances, in the context of globalization, socio-environmental problems are amplified, with social inequalities and environmental degradation worsening worldwide, and the ecovillages’ movement emerges in response. While a “North in the South” is configured by a reduced number of privileged people, and a “South in the North” is constituted by a growing marginalized contingent, the current levels of socioeconomic inequality are close to those registered at the beginning of the twentieth century, at the height of Western imperialism, with the richest 10% holding 78% of global wealth and half of the population holding only 2% of it, as reported by *The World Inequality Report 2022* (Chancel et al. 2022). According to this source, the overcoming of twenty-first century challenges, including the climate emergency and the massive biodiversity loss, will not be possible without addressing such socioeconomic inequality levels. The *Global Risk Perception Survey* published this year by the World Economic Forum (World Economic Forum - WEF 2022) also emphasizes global inequalities and polarization, threats to livelihoods, and mental health deterioration as the main challenges in the short term, while it refers to failures in climate actions and noncompliance with the Paris Agreement (United Nations – UN 2015) as the greatest long-term global threats.

Thus, on the intersectionality of the convergent *polycrisis* (Morin and Kern 1999) — ecological, climatic, economic, social, ethical, political, psychological, and so on — the limitations of the hegemonic societal model to address such complexity are revealed. Therefore, the search for alternatives to face these interconnected challenges deserves broader analytical scrutiny from multiple angles and perspectives over the usual power devices and the meaning of development itself. As argued by post-development scholars, like Escobar (1995) and Sachs (1992), development, as we know it, is unjust, never worked, and at this point has clearly failed.

Regarding the “top-down” approaches to the ongoing debate on the 2030 Agenda (United Nations – UN 2015), a global action plan for people, planet, prosperity, and peacebuilding, it still focuses on the ecological, economic, and social tripod of the polysemic notion of sustainability (Irving and Oliveira 2012; Irving 2014). Otherwise, the additional fourth “hidden” and subjective dimension of culture is also considered fundamental in “bottom-up” perspectives from community-led initiatives at the local level of ecovillages (Liftin 2014). Embodying human values and worldviews that reflect more sustainable lifestyles, ecovillages can contribute to reducing negative environmental impacts and producing positive ones as indicated by several studies (Daly 2017; Sherry 2019; Roysen and Mertens 2019; Roysen et al. 2021), including the environmental impact assessment conducted by the Global Ecovillage Network (Global Ecovillage Network - GEN, 2022). Thus, they can contribute to promoting regenerative paradigm shifts concerning the capitalist production and consumption model, disseminated under the aegis of development.

Dawson (2013) emphasizes the resumption of self-management over common goods and the cultural and economic renewal with respect to the Earth’s vital cycles as the guiding premises of community-led practices in ecovillages. The author also stresses that both intentional and traditional communities criticize the current development paradigm, which links economic growth to well-being. As mentioned by Liftin (2014, p.190), “these communities have been creating parallel structures for self-government within the prevailing social order while demonstrating how to live well with less.” For some traditional communities, becoming an ecovillage also means claiming spiritual and cultural integrity as well as safeguarding respect for communal tradition and self-determination threatened by colonization and modernization (Dawson 2006; Simas 2013).

Although feasible solutions and politics for a just and sustainable transition to a low-carbon society can be inspired by the heterogeneous grassroots innovations of ecovillages — as signalized by the image of the Findhorn Ecovillage featuring the cover of the Third Part of the Sixth Assessment Report of the International Panel for Climate Change (Mitigation of Climate Change ) (Intergovernmental Panel on Climate Change - IPCC 2022b) — it is still important to consider the political ideology concerning cultural values on the ecovillages’ approaches. This is because the alignment of some contesting initiatives with the current dominant societal frameworks, which do not seek the structural transformation of society (such as the economic or political-institutional order), can represent a “functional adaptation” to the capitalist game rules (Silva 2014). Under the aegis of capital, the leading role of counterculture movements can be co-opted by the market logic, interpreted in many situations as the only and inevitable pathway to solve development problems, including socio-environmental issues. Thus, the oppositional trait of counterculture movements can be lost by becoming innocuous for the needed social transformation and, also, convenient with other “sustainabilities”: those of the capital and the *status quo* (Leff 2006; Silva 2014).

Therefore, due to frustrations with the unfulfilled development well-being promises, in the current challenging times, new possibilities are open to alternative ethical-political projects, based on more inclusive and diverse proposals (Hidalgo-Capitán et al. 2019; De la Cadena and Blaser 2018). In different sociocultural contexts from those that originated the ecovillage movement, in Latin America, for example, ecovillages have been increasingly associated with ancestral cosmogonies of the Andean and Amazonian regions and the biocentric societal paradigm of *Buen Vivir*, connected to the recognition of the rights of nature as a living being (Acosta 2010; Walsh 2010; López and Prada 2015; Chaves et al. 2017; Gudynas 2011, 2019; Muñoz-Villarreal 2018). The commitment of the ecovillage movement to community-led alternatives based on different worldviews and horizontal relationships, in this way, tends to strengthen their countercultural nature, enabling emancipatory organizations and new productive arrangements and territorialities to emerge (Silva 2014).

Meanwhile, in the Brazilian context, for instance, as highlighted by Dias et al. (2017), most communities interlinked to the ecovillage movement, resemble those of the “Global North,” and composed of privileged social groups with a homogenous profile, which is mainly middle or upper middle class, ethnically “white,” with higher education levels. Despite the frequently expressed interest in diversity, “apparently, there is no significant link between traditional communities and the ecovillage movement in Brazil, although it is possible to consider them ‘entities’ that are alike in many aspects” (Dias et al. 2017, p. 83). Maybe this shows that, in some ways, there is room for future agencies and straightening relationships between ecovillages and other grassroots community-led alternatives in the “Global South” at the local and regional levels, as illustrated by the confluence between the Instituto Biorregional do Cerrado (IBC), an Ecovillage and Permaculture Center, and the largest Quilombola^4^ territory in Brazil called Kalunga. Both are located in a Brazilian rural area in the Cerrado^5^ biome, a biodiversity world hotspot endangered by the advance of the agribusiness frontier for large-scale monoculture commodities production (mostly soybean and corn) and are together engaging in the local political struggles in the decision-making arena, resisting along 20 years, against the construction of a hydroelectric power plant in the traditional territory (Roysen and Schwab 2021).

## Transition horizons: post-development, interculturality, and the confluence within the *pluriverse* of community-led alternatives

Based on the previous discussion, whereas different social groups face socio-environmental collapses and the convergence of multiple crises in the frontline of their territories, they also promote propositive movements and innovative political practices of collectivization and localization, germinating transformative alternatives.

Looking into ecovillages as laboratories of innovations and social technologies for sustainability, developing and testing solutions adaptable to each territorial context at the microscale of the communities open up a range of new possibilities that may be appropriated by the broader debate on building more sustainable futures (Seyfang and Smith 2007; Norberg-Hodge 2019; Roysen and Schwab 2021). On the other hand, several indigenous, black peoples, rural smallholders, and other local communities are working toward autonomy and self-determination, solidary economy, and reclaiming human, territorial, and nature rights. Along with sustainable food production through agroecology and agroforest restorative practices, rural development based on sociobiodiversity conservation and decentralized governance for economic democracy, they are leading innovations in several fields, also inspiring sustainable transition horizons (Escobar 2018; Kothari et al. 2019; Kothari 2021; Ferreira and Felício 2021; Lang 2022).

By developing feasible solutions to many of the contemporary challenges, this *pluriverse* of community-led alternatives should deserve greater attention from public policies, especially the redistributive ones, at a time when humanity is facing the risk of an average global temperature increase of 2.7 °C by the end of this century (Intergovernmental Panel on Climate Change - IPCC 2021). In this sense, as a “red alert for humanity,” the first part of the Sixth Assessment Report of the Intergovernmental Panel on Climate Change (The Physical Science Basis) (Intergovernmental Panel on Climate Change - IPCC 2021) emphasizes that a radical civilizational transformation is needed in this decade to avoid unpredictable changes, in the case of an average increase in temperature greater than 1.5 °C. However, even if the carbon emission neutrality and the net-zero goals of the Paris Agreement are achieved, according to the same report, this increase is most likely to reach 2.2 °C, which further reinforces the need for transformative transitions to radical systemic changes.

Furthermore, to face the panorama of uncertainties regarding climate change adaptation (Intergovernmental Panel on Climate Change - IPCC 2022a) and mitigation strategies (Intergovernmental Panel on Climate Change - IPCC 2022b), as well as for targeting the 17 SDGs of the 2030 Agenda (even considering the contradictions of this global agreement), it seems essential to strengthen and expand the bonds of collaboration with those social actors that are regenerating life conditions based on communal micropolitics. But even though the role of civil society is crucial for resilience and territorial autonomy, from a post-development perspective (Sachs 1992; Escobar 1995), broader alliances with governments and other decision-makers are determinants to address climate justice, eradicate poverty, and restore the natural environment, safeguarding human and planetary needs.

In order to achieve these transformations, for instance, ancestral knowledge and principles of conviviality (Illich 1973), like *Buen Vivir* (Acosta 2010; Walsh 2010; Gudynas 2011), can confluate with selective modern dimensions or institutions “in a self-determined way,” as shown by Lang (2022) in a study conducted with the Kayambi Kichwa people around their engagement in municipality politics in the northern Ecuadorian Andes. In this way, bringing theory into practice implies reducing the gap between the abstract and ambiguous concepts of sustainability and the concrete transformations that are being undertaken at the territorial level. For that, a real predisposition to an intercultural dialogue of knowledge and practices is fundamental, which implies tensions, conflicts, and pacts based on ethical relationships of respect for differences (Leff 2006; Santos 2007; Cusicanqui 2018; Walsh 2009).

It is also important to stress that the lack of openness to the other’s knowledge can hinder potential dialogues even between the social movements themselves, as well as between the ecovillage movements and other counter-hegemonic community-led alternatives, such as those led by the landless rural workers, indigenous peoples, or connected to urban and agrarian land reforms, for example. In this sense, agencies and network collaboration based on territorial articulations tend to be of great value, especially if accompanied by an ethical-political perspective committed to a new sustainable order (Porto-Gonçalves 2002; Haesbaert 2007; Silva 2014). According to Leff (2006, p. 232), “such transformation processes will imply the encounter of multiple rationalities, something much more complex and complicated, but more viable as a sustainability strategy than what dictates the market.”

Understood as an ideology and social vision intrinsic to the ideals of modernization, development holds western economic structure and society as a universal model for others to inevitably follow and emulate (Sachs 1992). Like a “ruin in the intellectual landscape,” as highlighted by the leading post-development scholar Wolfgang Sachs (1992), it is time to “dismantle this mental structure.” So, to decolonize the imaginaries from development concepts, post-development theorists promote more plural ideas about well-being based on interculturality (Walsh 2009). Instead of an unsustainable and also ineffective model of industrialization (due to their ignorance of the local, cultural, and historical contexts of the peoples to which they are applied), cross-cultural dialogues and interactions move beyond a passive acceptance of the multiple coexisting cultures to promote a set of relations grounded in values of mutual respect between indigenous and western ideals (Lang 2022).

**Fig. 1:**
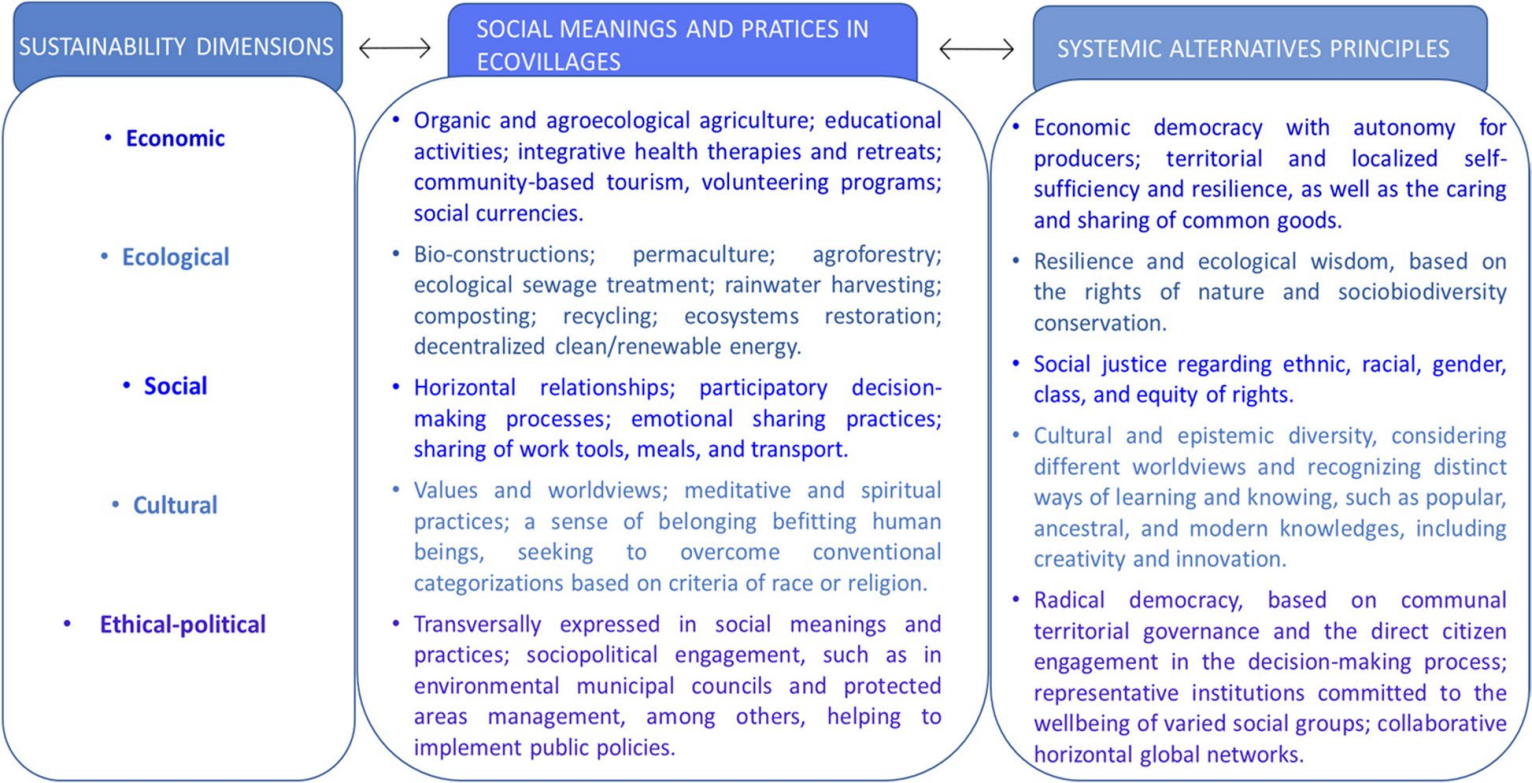
Synthesis matrix to articulate sustainability dimensions, social meanings and practices in ecovillages, and the guiding principles of systemic alternatives. Source: developed by the authors based on Roysen et al. (2021) and Kothari (2021)

To illustrate and push forward this debate on the possible confluences^6^ within the *pluriverse* of the community-led alternatives, as horizon solutions for a just and sustainable transition, Fig. 1 didactically articulates the dimensions of sustainability, social meanings and practices in ecovillages, and the guiding principles of systemic alternatives, based on a post-development and intercultural perspective of analysis.

Reflecting on the social meanings and practices in ecovillages, connected to the sustainability dimensions, it is possible to recognize that they align with systemic alternative principles in many ways, regarding the economic, ecological, social, cultural, and ethical-political aspects. This is because the *pluriverse* of community-led alternatives engages in an important variety of projects and actions connected to the commitments of ecological integrity, social equity and justice, meaningful participation, collective responsibility, cultural diversity recognition, and solidarity values (Escobar 2018; Roysen et al. 2021; Kothari 2021; Solón et al. 2019).

Some of these processes concern subsistence economies and local commerce, alternative health practices, oral and hands-on learning, and other practices, mostly considered outdated or “primitive” nowadays by the modern-development mindset. Otherwise, from a post-development and intercultural perspective, based on the *Buen Vivir* paradigm, within which nature and society are inseparable and the utmost respect for *Pachamama* (Mother Nature) is required, they reveal that human well-being and intergenerational sustainability can be achieved without endangering Earth’s biodiversity, including ourselves.

This resignification of well-being itself, not as a result of increasing material accumulation but as a way to safely meet livelihood needs, access satisfying learning and working opportunities, and share good social relationships, without generating ecological degradation and social exclusion, leads to profound cultural values review, regarding the relationships with oneself, the others, and the whole nature. This does not imply an unconditional acceptance of traditions or “a return to the past” but a recognition of the creative potential which results from mixing traditions and innovations linked through values that inspire better forms of conviviality (Illich 1973). On these bases, as extensively discussed by De la Cadena and Blaser (2018), Kothari, Escobar, and several authors (Kothari et al. 2019), rooted in ethical principles of interconnectedness and nurtured by the rich global biocultural heritage, different life territories integrate a *pluriverse* of possibilities based on territorial autonomies and in arrangements among local economies, configuring a “global tapestry of alternatives” (Kothari et al. 2019) to ailing contemporaneous lifestyles.

In this debate, it is fundamental to recognize the contribution of the daily life struggles of vulnerable social groups mobilized by environmental justice and land rights to reconfiguring the geopolitics of knowledge. By challenging the abstract theories of the dominant Eurocentric episteme divorced from territorial realities, they turn these “living utopias” possible to emerge, questioning and refusing the colonial, racist, patriarchal, and anthropocentric premises, intrinsic to modern hegemonic social meanings and practices underlying current power structures (Dilger et al. 2016; Dinerstein 2017; Quijano 2000; Walsh 2010; Ferreira and Felício 2021).

Therefore, overcoming conventional sustainability perspectives implies the recognition of other worldviews and environmental rationalities (Leff 2006), diverse in the ways of being-knowing-living. While instrumental narratives based on a fragmented and disenchanted worldview lead to the present scenario of uncertainties, plural narratives can contribute to re-enchantment, “suspending the sky” and “postponing the end of the world” (Krenak 2019). Consequently, shedding light on the *pluriverse* might inspire hope and expand horizons for regenerative possibilities toward desirable futures. Nature-based solutions to face climate change and biodiversity loss, thus, require narratives that transcend the disjunctive and reductionist thinking and binary splits between nature and culture, like *Buen Vivir*. From a systemic perspective, climate actions should result from a deep process of sociocultural transformations, presupposing the overcoming of the coloniality intrinsic to the materialist and accumulative logic (Quijano 2000).

From this analytical perspective, post-development proposals from the “Global North,” such as deep ecology, degrowth, and ecofeminism (Beling 2019; Kothari et al. 2019), seem to converge with the Epistemologies of the South (Santos 2007). Therefore, some of the results from the industrialization process and technological development (such as information and communication technologies) can contribute forward to the emergence of plural transition horizons, converging on complementary relations in a synthesis between “organic and synthetic knowledge”^7^ (Santos 2015).

As a post-development (re)existence strategy, built in the confluence of indigenous cosmovisions and the critical thinking against extractivism, through the dispossession of territories in Latin America (Svampa 2019), for instance, *Buen Vivir* is becoming an emergent platform for new socio-political and environmental praxis (Acosta 2010; Walsh 2010; Gudynas 2011, 2019; Lang 2022). In this sense, as an inspiring “North-South” confluence, ecovillages can represent a translocal empowerment strategy through which the meanings of “good coexistence” can recreate living worlds, in tune with the triune dimensions of cultural identity, social equity, and ecological sustainability of the *Buen Vivir* communal ethics.

## Concluding remarks

A context of multiple crises arises from sociohistorical processes concerning the current development model. For this reason, the ethical-political debate on post-development scrutinizes the notion of development itself, inspired by territorialized and community-led alternatives that can promote more sustainable, resilient, and equitable ways of being-knowing. Although diverse in socio-environmental practices and meanings, the *pluriverse* of community-led alternatives is oriented by premises that rely on safeguarding the “commons” governance based on the recognition of the profound ties of interdependence between humans and nonhumans, which transcend the modern utilitarian rationality of the capitalist, colonial, patriarchal, and anthropocentric world-system view (Porto-Gonçalves 2002; Escobar 2018; Kothari et al. 2019; Lang 2022).

In this regard, by experiencing sensitive pathways to respectfully inhabit the planet, ecovillages can be understood as “living laboratories” (Santos-Júnior 2016; Mattos 2018) where the integration of the various areas of human life (education, health, economy, construction, agriculture, energy, management, politics, etc.) is often conceptualized around four interconnected dimensions: ecological, social, economic, and cultural/spiritual (Gaia Education 2005). Nevertheless, it is essential to understand ecovillages as a process in which collective learning can be continuously (re)constructed, and not as a pre-formatted small-scale model of society. Primordial learning from these experiences, for instance, relies on ethical-political principles guided by diverse epistemological and ontological basis. This is because broader and inclusive cross-cultural alliances with diverse and marginalized social groups, together with the empirical knowledge and innovations interconnected to Earth’s life cycles, can contribute to expanding transition horizons for resistance and (re)existence, facing the complex contemporary challenges.

Therefore, in contrast to current catastrophic projections, resulting from the unlimited and unidirectional progress beliefs, richer subjectivities emerging from these community-led confluences help to decolonize imaginaries and deconstruct rooted development premises, expanding the fields of possibilities for desirable futures and inspiring public policies, from the very basis of society, to address the Paris Agreement and the 2030 Agenda commitments. Rather than becoming a radically opposing ideology, the *Buen Vivir* approach in these cases provides opportunities to meet the core aims of sustainable development through the *pluriverse*, as possible alternatives that go beyond the conventional anthropocentric development model.

Whereas there is now an impetus toward alternatives to face the countless ongoing uncertainties and predicted collapses — materialized by the tragic context of the Covid-19 pandemic, the climate emergency, and the geopolitical insecurities — it is essential to deploy “colorful parachutes” (Krenak 2019) and to be open to the inventive capacity of the Earth’s intertwined living intelligences.

## Data Availability

Not applicable

## Footnotes

1 Minoritarian social groups do not refer to an amount but to the discrimination suffered by some social groups such as indigenous, native, women and poorer people due to racial, socioeconomic, cultural, or gender issues, highlighting their insufficient representativeness in power spaces.

2 Different connotations and definitions regard traditional communities. In the Brazilian legislation, they are conceived as “culturally diverse groups that recognize themselves as such, that have their own forms of social organization, that occupy and use territories and natural resources as a condition for their cultural, social, religious, ancestral, and economic reproduction, using knowledge, innovations, and practices generated and transmitted by tradition” (Brasil 2007).

3 Between the privileged and the marginalized by the capitalist system

4 Traditional, legally recognized, Afro-descent self-sufficient communities that were created in hidden remote rural areas by ancestors escaping from slavery, most of them with some help of native indigenous people, whose both ancestral lifestyles are closely connected to their environments (Brasil 2007).

5 Cerrado is the second largest biome of the South American continent, considered the world’s most biodiverse savanna. Known as “the cradle of the waters” in Central Brazil, it “gives birth” to two-thirds of the hydrographic basins in the Brazilian territory (Instituto Brasileiro de Geografia e Estatística – IBGE 2007).

6 Confluence is designed as “possible connections for strengthening trajectories, a lesson learned from the waters as they expand their courses in flow and power when they meet” by Antônio Bispo dos Santos, a Brazilian intellectual of a Quilombola traditional community (Santos 2015). The environmental sociologist Ashish Kothari also adopts the term in a similar sense, in the context of the “Global Tapestry of Alternatives” and “Radical Ecological Democracy” networks (Kothari et al. 2019).

7 According to Santos (2015, p. 101), “there is an organic knowledge and a synthetic knowledge. While organic knowledge develops being, synthetic knowledge develops owning. We are operators of organic knowledge and colonialists are operators of the synthetic.”
